# Darwinian Evolution of Intelligence

**DOI:** 10.3389/fbinf.2022.838420

**Published:** 2022-05-27

**Authors:** J. C. Phillips

**Affiliations:** Department of Physics and Astronomy, Rutgers University, New Brunswick, NJ, United States

**Keywords:** neural network, amino acid sequence, thermodynamic and phase properties, natural selection 3, sliding window

## Abstract

Intelligence is often discussed in terms of neural networks in the cerebral cortex, whose evolution has presumably been influenced by Darwinian selection. Here we present molecular evidence that one of the many kinesin motors, KIF14, has evolved to exhibit a special feature in its amino acid sequence that could improve neural networks. The improvement is quantified by comparison of NIF14 sequences for 12 species. The special feature is level sets of synchronized hydrophobic extrema in water wave profiles based on several hydropathic scales. The most effective scale is a new one based on fractals indicative of approach of globular curvatures to self-organized criticality, which summarizes evolutionary trends based on intelligent design.

## Introduction

There are at least 14 kinesin motor families (Lawrence et al., 2004; Miki et al., 2005), and the mechanics of the hand-over-hand kinesin step dragging cargo along tubulin have been well studied (Yildiz et al., 2004; Carter and Cross, 2005; Block, 2007). A bioinformatic survey of 1,624 putative kinesins identified three families that are very widespread among species: KIF 1, 5, and 14 (Wickstead et al., 2010) (see their Figure 1). According to Uniprot, KIF1 provides “anterograde axonal transport of synaptic vesicle precursors,” KIF5 is “required for slow axonal transport of neurofilament proteins,” and “during late neurogenesis, KIF 14 regulates the cerebellar, cerebral cortex and olfactory bulb development through regulation of apoptosis, cell proliferation and cell division (by similarity)”. Other kinesins are less common and appear to be less involved in the complex task of neural network assembly and maintenance. All of the families except KIF14 walk towards the tubulin plus end (Miki et al., 2005). This means that KIF14 can make refinements in neural network tubulin end structures created by KIF 1, 5, and possibly other kinesins.

**FIGURE 1:**
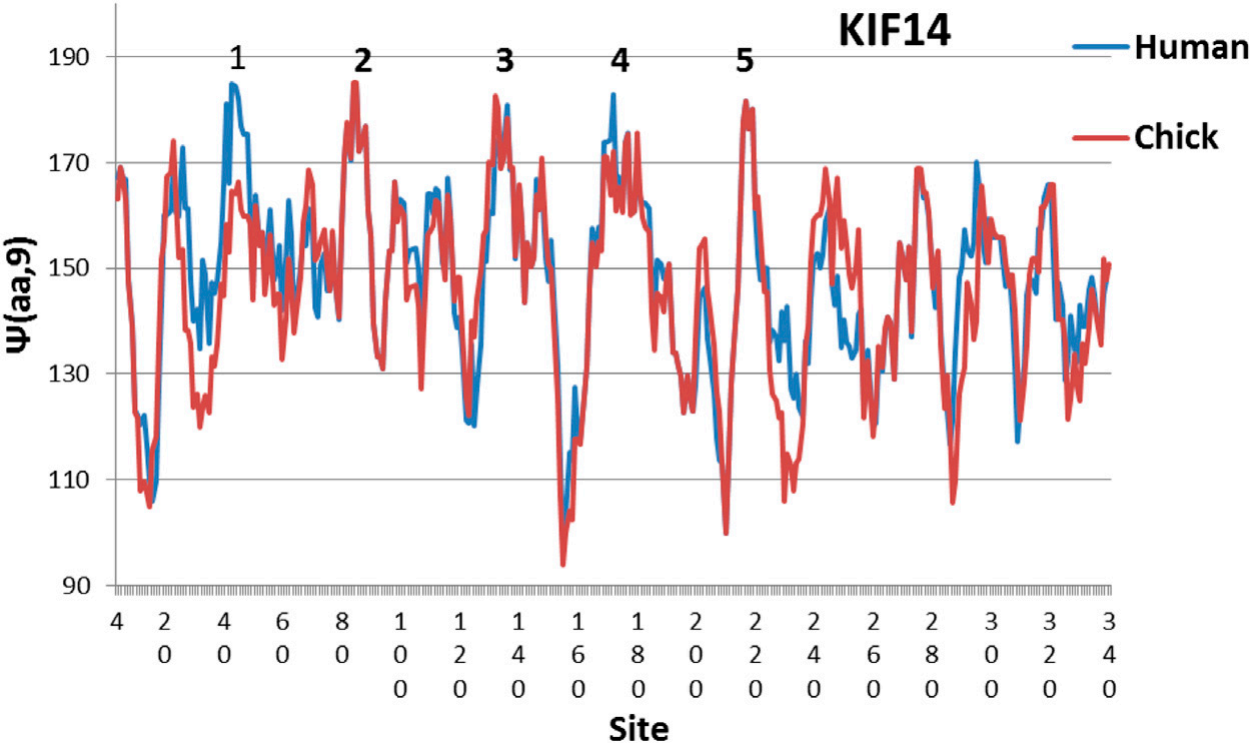
Evolution leveled hydrophobic peaks 2, 3 and 5 in chicken KIf14 motor profiles with W = 9, and further evolution added level peaks 1 and 4 in human. The reader may also observe partial leveling from chickens to humans of the five lowest hydrophilic minima near 10, 150, 210, 230 and 290. The MZ scale is used here (Moret and Zebende, 2007; Phillips, 2020). In other proteins the number of level peaks is usually only three (Phillips, 2009)

Many distinctive features connecting protein sequences to their functions and evolution are related to globular-shaping water waves obtained by thermodynamic scaling. The general method used here is only 12 years old, and is little known. It is reviewed in our earlier companion article on dynein (Phillips, 2020).

Phylogenetics counts numbers of identical or similar amino acids at specific sites using BLAST, and it is limited by the restriction to single sites. There is an alternative to the single site methods, which has Darwinian selectivity as an implicit feature, as corroborated by the identification of universal, amino-acid specific self-organized criticality in the solvent-accessible surface areas (SASA) of >5,000 protein amino acid segments from the modern Protein Data Base (Moret and Zebende, 2007; Phillips, 2009). The lengths of the small segments L = 2N + 1 varied over a wide range from 3 to 45, but the interesting range turned out to be M< = 9 ≤ L ≤ 35 = M>. Across this range they found linear behavior on a log-log plot (a power law, hence self-similar) for each of the 20 amino acids centered on a given segment

logSASA(L)∼const−Ψ(aa)logL (9≤L≤35)

Here Ψ(aa) is a hydropathicity parameter. It arises because the longer segments fold back on themselves, occluding the SASA of the central aa. The most surprising aspect of this self-similar folded occlusion is that it is nearly universal on average across the proteome. Protein folding has been the subject of hundreds of thousands of studies of specific structures, and it is indeed remarkable that Ψ(aa) is almost independent of the individual protein fold. This is a dramatic demonstration of the power of Darwinian selectivity involved in aqueous shaping of globular proteins, as discussed in detail elsewhere (Phillips, 2016). Moreover, the segmental character of the new scale (Moret and Zebende, 2007; Phillips, 2009) has a Darwinian echo: for each protein family one can identify an optimized sliding window width W*, over which Ψ(aa) is best averaged to maximize the resolution of evolutionary improvements; this averaged profile is denoted by Ψ(aa,W*). Profiles of Ψ(aa,W*) display the functional features that are being optimized by evolution, often involving modular (segmental) exchange (Phillips, 2009).

## Results

Evolution has leveled sets of (more often) hydrophobic and (less often) hydrophilic extrema in profiles of many proteins (Phillips, 2009). Such leveling optimizes (synchronizes) protein multi-domain hydrodynamics, in accordance with Sethian’s level set hydrodynamic theory (Saye and Sethian, 2011; Phillips, 2016; Allan and Phillips, 2017) Protein-protein interactions are complex, and level sets are not the rule. However, when they do occur, they can be used to identify more easily quantified protein dynamics, and monitor functional evolution. In contrast to molecular dynamics simulations, it is easy to analyze profiles even of very large proteins of more than 1,000 amino acids (Phillips, 2016) near equilibrium and find level sets. Here we inspect KIF 1,5 and 14 motor profiles (∼350 aa) and find that there are no level sets in the extrema of KIF1 motor profiles, while the KIF14 human motor profile exhibits striking level sets of hydrophobic extrema. KIF5 is an intermediate case, with little evolution and only some level set aspects, so we focus on KIF 14 and its substantial evolution in late species.

The optimal value of the sliding window width W* can be determined in different ways, depending on the protein family under study. Here we studied graphs of human profiles with W ranging from 7 to 21, which showed the optimal human value is W* = 9. In general smaller values of W give higher resolution, while larger values of W are more likely to treat segmental dynamics more accurately. In addition to the MZ 2007 fractal scale, which is consistent with self-organized criticality (Phillips, 2009), we also used the standard 1982 hydropathicity scale (KD), which gave weaker results (Niu et al., 2006). Note that W = 9 is the lower limit of the fractal range spanned by the MZ areas; smaller values of W are better treated with the first-order KD scale. It appears that neuronal dynamics are on the second-order side of the edge between first- and second-order interactions.

The effects of evolution on KIF14 are illustrated in Figure 1, which compares human and chicken profiles. The chicken level set has three peaks which grow to five peaks in human. Alternatively, one can find the deviation from the mean for the five highest hydrophobic peaks. This is smallest in humans, and it increases for earlier species. The mean deviation of the highest five peaks for human KIF1 is 10.8, or 7 times larger than human KIF14 (practically an empty level set). (KIF1 is the most common kinesin, and supports growth of cells in many tissues.) Altogether this is good circumstantial evidence that KIF14 is important to building and refining neural networks.

Numerical results for mean deviations of the highest five peaks of many species are shown in Table 1, together with the number of BLAST positives that differ from human. The reader may judge which column more accurately reflects relative intelligence. The general principles of self-organized criticality have been known for decades (Kyte and Doolittle, 1982), but physical examples have been limited (Mandelbrot, 1982). The emergence of fractals in protein surface areas (Moret and Zebende, 2007) suggests many genomic applications, as discussed in earlier articles (Phillips, 2009; Phillips, 2016; Phillips, 2020).

**TABLE 1:** Deviations from the mean for each species by the five highest hydrophobic peaks in the KIF14 motor profiles. Also shown are the number of BLAST positives that differ from human. The small values of mean deviation for whale, zebra fish, and wild turkey may represent the effects of extreme environmental conditions. Such anomalies could be minimized in canine data (dogs and humans share similar environments).

Species	Mean devia.	Positives
Human	1.50	0
Whale	1.78	30
Zfish	1.88	40
W Turkey	2.52	48
Elephant	2.55	10
Mouse	2.73	17
Pol. Bear	2.76	10
Rabbit	2.94	15
Fox	3.06	14
Horse	3.42	21
Chicken	3.91	45
Mole Rat	4.10	12

**FIGURE 2:**
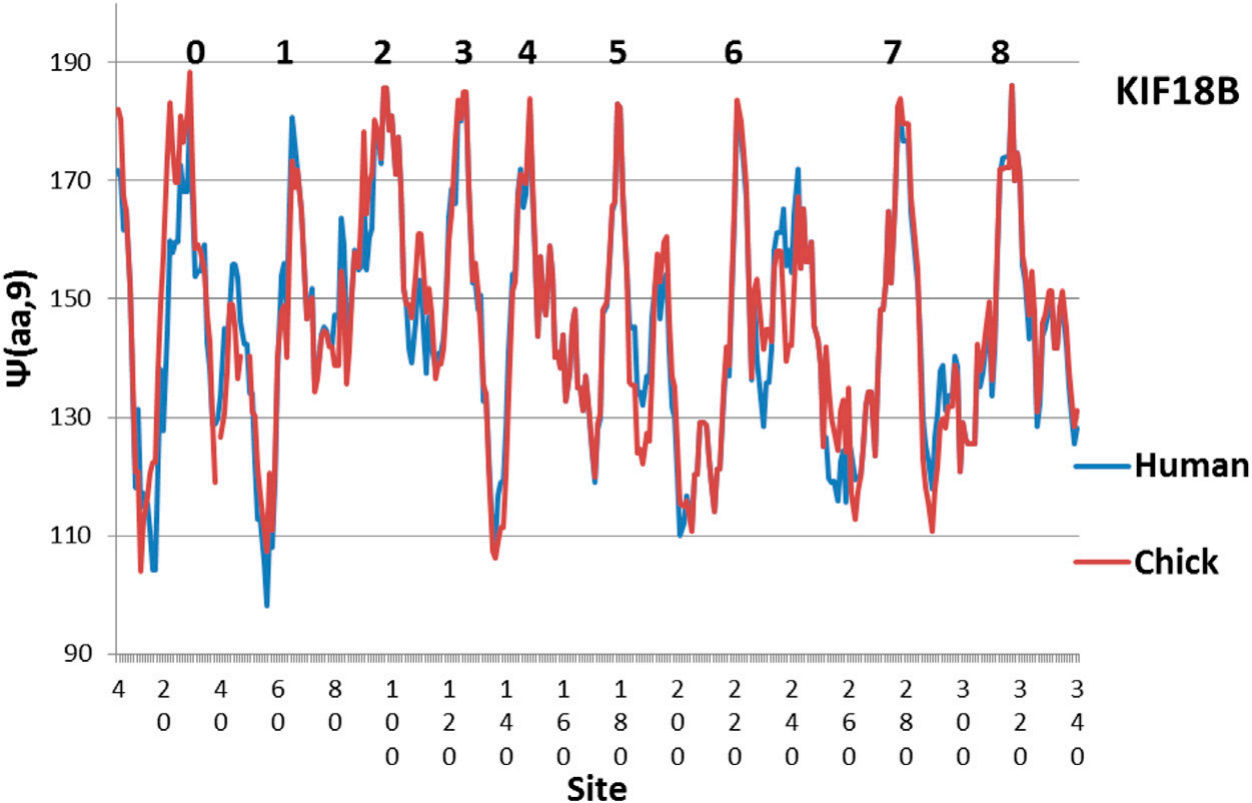
The large differeences between Human KIF18B suggest that Chicken KIF 18B may perform a special function, as its hydrophobic extrema are much more level (MZ scale).

**FIGURE 3:**
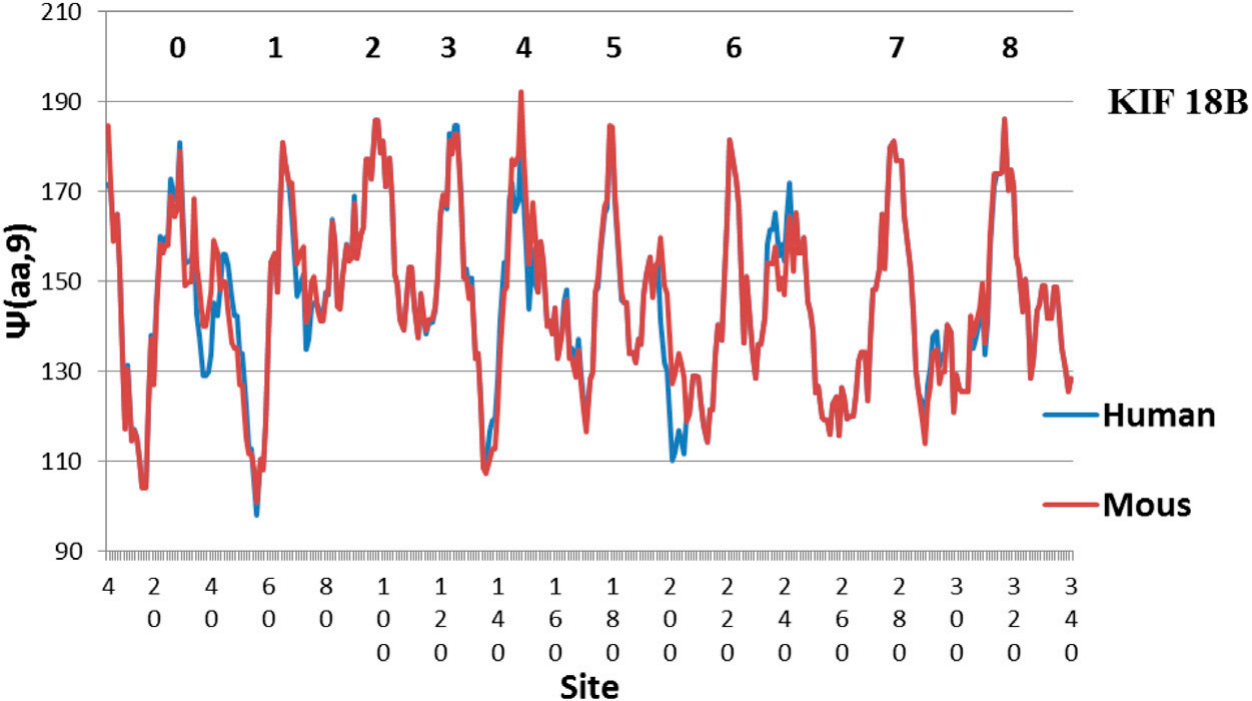
Compared to Figures 1, 2, Mouse KIF18B is now similar to Human KIF18B. The main difference is Peak 4, which is more level in Human (MZ scale).

**FIGURE 4:**
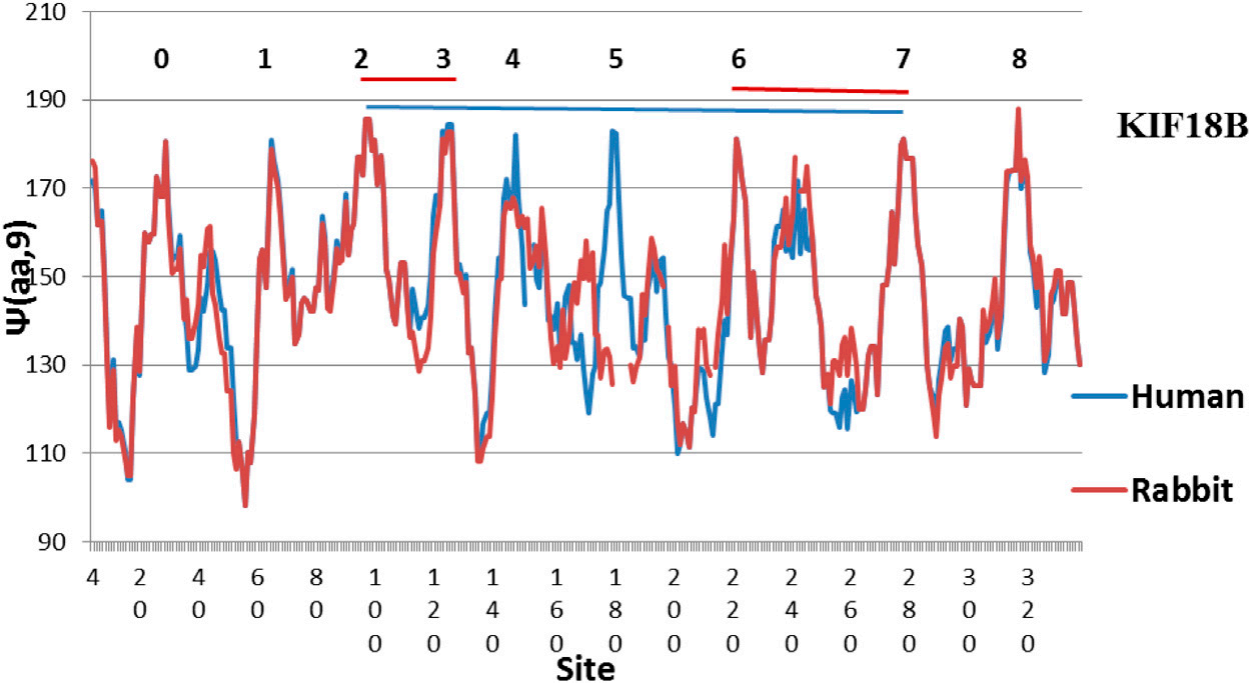
There are still diffences in KIF18B between Human and Rabbit, with the Human peaks more level (MZ scale).

**FIGURE 5:**
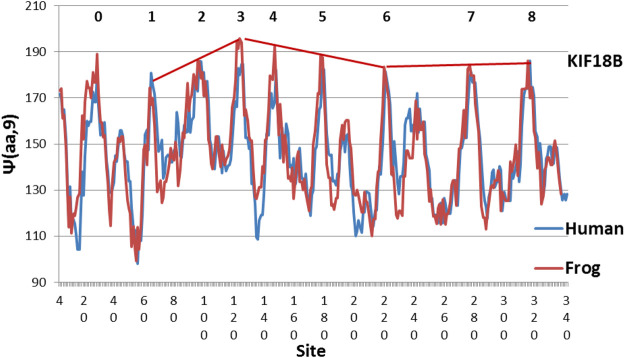
Frog KIF18B is an extreme example, with hydrophobic peak structure peaking at Peak 3 (MZ scale).

The evolution of KIF18B is shown in Figure 2. Here similarly level peaks are absent from the Human profile, but present in the Chicken profile! So far all of the few studies of KIF18B have used mammalian samples only. It would be interesting to see if chicken KIF 18B behaved differently. Interesting variations are seen in other species in Figures 3–5.

Domestic dog breeds are characterized by an unrivaled diversity of morphologic traits and breed-associated behaviors resulting from human selective pressures. A recent survey of whole genome sequences of 722 dogs found 16 phenotypes that explain greater than 90% of body size variation in dogs (Albert and Barabasi, 2001). An even larger survey of 14,000 dogs with owner - assessed traits was connected to 131 single nucleotide polymorphisms, primarily in brain genes (Plaissais et al., 2019). To go beyond phenotypes and traits to assess intelligences of different breeds, one needs specific proteins. The ultimate mechanism for dynein and kinesin molecular motor interaction with the extended axons of neurons could involve as many as 1,500 mitochondrial DNA proteins ((Schwarz, 2013; MacLean et al., 2019), Wiki). Here we have presented evidence that KIF14 is evolutionarily critical for neural networks and deserves special attention. If KIF14 motor sequences could be extracted from the canine data base, a correlation with canine intelligence variations could be discovered. In less than 20 years, the number of dogs in America has increased from 65 million to 90 million, so the discovery of such a connection would be of great interest. One could also study genomic differences between working and conformational border collies, as they could be exceptionally large for genes associated with KIF14 (see https://en.wikipedia.org/wiki/Border_Collie#ISDS_sheepdog_trial).

Here we have emphasized small differences in healthy neural networks as a measure of intelligence. KIF14 mutations cause brain malformation and microcephaly (Fujikura et al., 2013; Johnston and Williams, 2016). The concept of self-organized criticality has been widely discussed for its implications for living matter (Makrythanasis et al., 2018). Fractals are keys to modern statistical mechanics. (Munoz, 2018), and nanoscale biological features are increasingly studied (Stanley and Taylor, 1994). It appears that the level of dynamical synchronization increases with the number of level edges, for instance three in CoV03, and four in CoV19 (Dawson and Yan, 2021; Phillips, 2021). The numbers of KIF14 synchronized edges of three in chicken and five in humans (Figure 1) are consistent with the concentration of KIF14 in the prefrontal cortex.

## Data Availability

The original contributions presented in the study are included in the article/Supplementary Material, further inquiries can be directed to the corresponding author.
